# Comment upon “Time to epinephrine treatment is associated with the risk of mortality in children who achieve sustained ROSC after traumatic out-of-hospital cardiac arrest”

**DOI:** 10.1186/s13054-019-2614-3

**Published:** 2019-10-29

**Authors:** Benjamin Post, Dominic Peter Douglas Nielsen, Anil Visram

**Affiliations:** Department of Paediatric Anaesthesia, Royal London Hospital, Whitechapel Road, Whitechapel, London, E1 1BB UK

Editor,

We read with great interest the recent article from *Critical Care* examining the use of adrenaline in paediatric traumatic cardiac arrest [1]. The role of vasoactive drugs in traumatic cardiac arrest remains unclear, and previous studies in adults have produced conflicting results [2, 3].

Whilst we found the results to be interesting, we have concerns about the applicability to our practice. Firstly, the two study populations, defined as haemorrhagic shock (HS) and non-haemorrhagic shock (non-HS), were classified retrospectively. The study’s results indicate that early administration of adrenaline may be deleterious in out-of-hospital cardiac arrest (OOHCA) secondary to HS, but not in non-HS. Indeed, the authors recommend: “… the initial classification of HS or non-HS is important when considering the early administration of epinephrine in children with traumatic OHCA.” In an emergency situation, the total volume of blood loss may not be clear until a period of stability is reached. Furthermore, cardiac arrest may occur prior to reaching a threshold value of blood loss > 30% of total blood volume. As such, we feel that it would be difficult in a dynamic clinical situation to accurately decide whether to administer adrenaline before or after the 15-min post-collapse time.

The study was conducted over a 12-year period during which time there were significant changes to the international consensus on management of major trauma [4].

We found the allocation of patients with spinal injury, airway injury, cardiac tamponade and tension pneumothorax into the “haemorrhagic shock” group surprising. In our institution, these injury patterns would not necessarily trigger the “major haemorrhage” pathways and we are interested as to why the authors grouped this cohort with patients with severe blood loss.

Fundamentally, we agree with the authors that “hemostasis, volume resuscitation and high-quality CPR” should be prioritised in major haemorrhage; however, we feel that the results of this study should be interpreted with caution and is unlikely to affect our current practice. This study, however, reaffirms the very poor prognosis associated with this catastrophic injury type [5].

## Data Availability

Not applicable.
